# Data for chitin binding activity of Moringa seed resistant protein (MSRP)

**DOI:** 10.1016/j.dib.2016.08.070

**Published:** 2016-09-07

**Authors:** Anudeep Sandanamudi, Kishan R. Bharadwaj, Radha Cheruppanpullil

**Affiliations:** CSIR-Central Food Technological Research Institute, Mysore − 570020

**Keywords:** *Moringa oleifera*, Seed protein, Resistant protein, Chitin binding protein

## Abstract

Chitin binding activity of moringa seed resistant protein (MSRP) isolated from defatted moringa seed flour was investigated in the present study “Characterization of soluble dietary fiber from *Moringa oleifera* seeds and its immunomodulatory effects” (S. Anudeep, V.K. Prasanna, S.M. Adya, C. Radha, 2016) [1]. The assay reaction mixture contained 0.4 mg/ml of MSRP and different amounts (20–100 mg) of chitin. MSRP exhibited binding activity over wide range of chitin concentration. Maximum binding activity was observed at 80 mg of chitin. The property of MSRP to bind chitin can be exploited for its purification.

**Specifications Table**

**Table:** 

Subject area	*Biology*
More specific subject area	*Protein binding activity*
Type of data	*Figure*
How data was acquired	*Spectrophotometer (Shimadzu UV-1601, Japan)*
Data format	*Analyzed*
Experimental factors	*Isolation and purification of MSRP was performed according to Anudeep et al.*
Experimental features	*All the incubation reactions were performed at room temperature; protein concentration was determined by Lowry׳s assay.*
Data source location	*Mysore, India*
Data accessibility	*Data is represented in this article*

**Value of the data**

• The data reveals that MSRP binds to chitin.
• Results indicated that 0.4 mg/ml of MSRP saturates at 80 mg of chitin.
• Present data shows the amount of chitin required to purify MSRP.
• Chitin binding property could be utilized to study the biochemical and structural properties of proteins.

## 1 Data

**Fig. 1:**
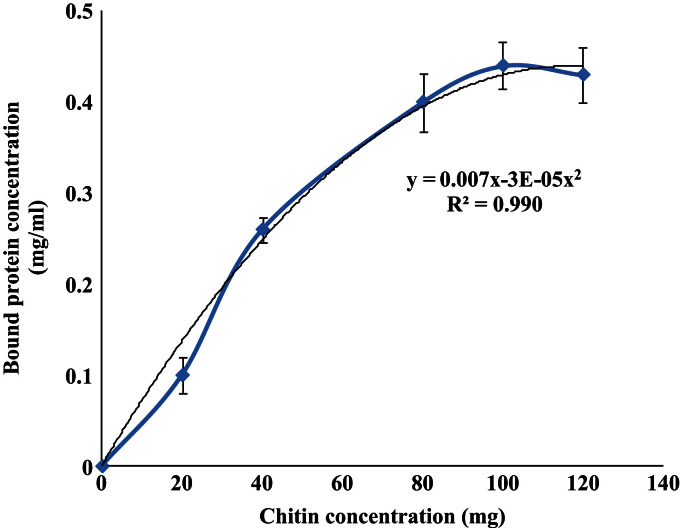
Chitin binding assay data for MSRP. The binding assay mixture contained 0.4 mg/ml of MSRP and from 20–100 mg of chitin. The graph shows the chitin binding capacity of MSRP; showing maximum binding at 80 mg of chitin for 0.4 mg/ml of protein. The experiments were performed in triplicates.

The data file determines the bound protein concentration (mg/ml). The amount of bound protein was calculated from the difference between the initial protein concentration and free protein concentration after binding. The data shown in Fig. 1 is the relationship between the weights of chitin in reaction mixture to the chitin bound protein (MSRP) concentration. The relationship follows a second degree polynomial equation.

$$B_{p}=0.007C_{h}-0.00003C_{h}^{2}$$

*B_p_*: Chitin bound MSRP concentration
*C_h_*: Weight of Chitin in reaction mixture

The goodness of fit of the above equation is 0.99 (or 99%)

## 2 Experimental design, materials and methods

### 2.1 Materials

Chitin was procured from HiMedia Laboratories Ltd., Mumbai, India. BSA was obtained from Sigma Chemical Company, St. Louis, MO, USA. All other chemicals and reagents were analytical grade.

### 2.2 Experimental design

#### 2.2.1 Isolation and purification of MSRP

MSRP was isolated and purified from defatted moringa seed flour according to Anudeep et al. [1].

#### 2.2.2 Chitin binding assay

Chitin binding assay was performed by incubating different concentrations of chitin (0, 20, 40, 80, and 100 mg) with 0.4 mg of MSRP in 1 ml of 0.25 M NaCl with occasional stirring for a period of 1 h at room temperature. The reaction mixture was then centrifuged for 20 min at 10,000 *g*. The supernatant containing free protein was collected and the protein concentration was determined by Lowry׳s assay [2]. The amount of bound protein was calculated from the difference between the initial protein concentration and free protein concentration after binding [3].
