# Supplementary material for: Data for chitin binding activity of Moringa seed resistant protein (MSRP)
Source: Data Brief. 2016 Sep 7;9:335–7. doi: 10.1016/j.dib.2016.08.070 (PMC5030337; doi:10.1016/j.dib.2016.08.070)
Supplement: Supplementary file 1 — Supplementary material [file mmc1.docx]

**CONFLICT OF INTEREST FORM**

**Title of the paper:** Data for chitin binding activity of Moringa seed resistant protein (MSRP).

**Authors:** Anudeep Sandanamudi, Kishan R. Bharadwaj, C. Radha

Behalf of all authors, the corresponding author of this paper declare that they have no conflict of interests.

Corresponding author

Dr. C. Radha

Senior Technical officer (2),

Protein Chemistry and Technology,

CSIR- Central Food Technological Research Institute, Mysore, INDIA
